# Difficult removal of a totally implantable venous access device 12 years after implantation: a case report and literature review

**DOI:** 10.1590/1677-5449.202500042

**Published:** 2025-05-30

**Authors:** Aymar Kassa Boukat, Mohamed Bhairis, Massine El Hammoumi, El Hassane Kabiri

**Affiliations:** 1 Mohammed V Military Teaching Hospital, Rabat, Morocco.; 2 University Mohammed V, Rabat, Morocco.

**Keywords:** implantable venous access device., cateter totalmente implantável.

## Abstract

Totally implantable venous access devices (TIVADs) are commonly used for prolonged intravenous treatment, particularly in oncology. Although removal is typically a straightforward procedure at the end of treatment, it can occasionally be complicated by adhesion of the distal end of the catheter to the intravascular wall. This rare complication is often associated with factors such as prolonged catheter dwell time, use of polyurethane material, and younger age at insertion. The technique used for removal depends largely on the degree of adhesion. We report the case of a 60-year-old woman with a TIVAD in place for 12 years for chemotherapy for breast cancer. Removal was challenging due to distal adhesion of the catheter but was ultimately successful following careful dissection and traction.

## Resumo

Cateteres totalmente implantáveis são dispositivos comumente utilizados para tratamento intravenoso prolongado, especialmente em pacientes oncológicos. Embora sua remoção geralmente seja realizada de forma simples ao final do tratamento, complicações podem surgir devido à adesão da extremidade distal do cateter à parede intravascular. Essa complicação rara está associada a fatores como tempo prolongado de permanência do cateter, uso de material em poliuretano e idade jovem no momento da implantação. A técnica utilizada para remoção depende, em grande parte, do grau de adesão. Relatamos o caso de uma mulher de 60 anos com um cateter implantado há 12 anos para quimioterapia contra câncer de mama. A remoção foi inicialmente desafiadora devido à adesão distal do cateter, mas foi bem-sucedida após dissecção cuidadosa e tração.

## INTRODUCTION

Removal of a totally implantable venous access device (TIVAD) is routinely performed at the end of a therapeutic protocol, except in cases of complications, patients lost to follow-up, or early/late removal in cancer survivors after remission. Although it is a relatively straightforward procedure, removal can occasionally present challenges, particularly when the distal tip of the catheter becomes adherent to the endovascular wall.^1^

Adhesion formation is associated with several factors, the most common being prolonged catheter dwell time.^2,3^ In such cases, the removal technique depends on the degree of adhesion of the distal end of the catheter to the endovascular wall, and may involve further dissection along the catheter tract, cutting of the catheter at its insertion point with the distal end left in situ, the use of a guidewire, or endoluminal dilatation.^2,4,5^

We report a case of difficult removal of a TIVAD with an aberrant course, in which the distal end of the catheter was adhered to the endovascular wall of the right brachiocephalic vein.

This research was conducted in accordance with the Helsinki Declaration.

## CASE REPORT

A 60-year-old woman with a history of infiltrating ductal carcinoma of the right breast underwent partial mastectomy followed by adjuvant chemotherapy. In 2012, a TIVAD was inserted via the left subclavian vein by the thoracic surgery team. Following remission, the TIVAD was left in place for long-term surveillance of the cancer.

**Figure 1:**
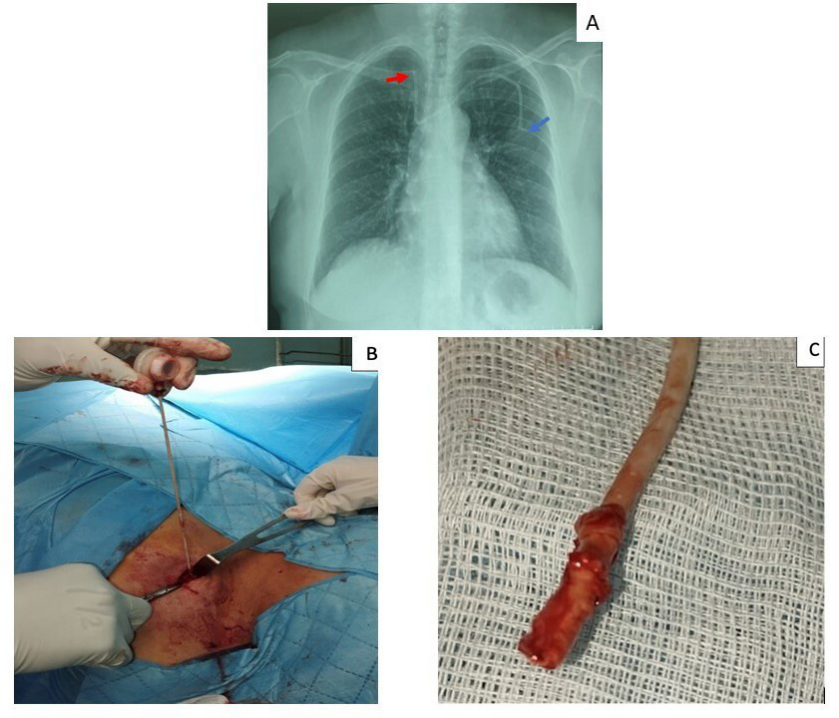
(A) Chest radiograph showing an aberrant course of the Port-a-Cath, with the distal end lodged in the right brachiocephalic vein (red arrow) and a slightly visible port chamber (blue arrow); (B) Traction of the polyurethane catheter, which was held in place by distal adhesion; (C) Fibrotic encapsulation in the distal end of the catheter.

Twelve years later, the patient was referred by the oncology department for removal of the TIVAD. On admission, physical examination revealed a subcutaneous port chamber connected to the proximal end of a catheter. The remainder of the examination was normal. Pre-ablation chest radiography (Figure 1A) revealed an aberrant course of the catheter, with the distal end lodged in the right brachiocephalic vein.

Surgical removal was performed under local anesthesia. A new incision was made over the old incision, followed by dissection of the fibrotic tissue surrounding the proximal end of the catheter and the port chamber, allowing for their release. During attempted removal of the catheter, resistance was encountered. Prolonged traction combined with careful dissection along the catheter tract – extending to the left subclavian vein – ultimately allowed for successful removal. The distal end of the catheter showed fibrotic encapsulation (Figure 1B and 1C), confirming its adhesion to the endovascular wall.

Its path was then closed with an X-stich using Vicryl 3-0, and after cleaning the implantation site of the extracted chamber, we closed the surgical wound. The postoperative course was uneventful. The patient was discharged 24 hours after surgery with a follow-up appointment scheduled for 10 days later. Wound healing was satisfactory, and no signs of local infection were observed.

## DISCUSSION

Central venous catheter removal is a relatively straightforward surgical procedure performed under local anesthesia. However, in rare cases, removal can be challenging. A retrospective analysis of TIVAD removals performed between 2003 and 2012 identified difficult removals in only 4% of cases, with 9% of these due to endovascular adhesion (n= 1306).^1^

Our case is consistent with the literature. Kabiri et al.^6^ and El Hammoumi et al.^7^ conducted two large series involving 970 and 1460 cases, respectively, of TIVAD implantation, primarily for chemotherapy in patients with cancer. Among observed complications, no cases of adhesion of the catheter’s distal end to the endovascular wall of the superior vena cava were registered.

Adhesion of the distal end of the catheter to the endovascular wall is associated with several factors, including younger age at insertion, a dwell time greater than 20 months, diagnosis of acute lymphoblastic leukemia, and the use of polyurethane catheters.^1,2^ In our case, the main contributing risk factors were prolonged dwell time of the Port-a-Cath – 144 months – and the use of a polyurethane catheter. These two factors were also reported by Mehra et al.,^3^ although their case involved a slightly shorter dwell time of 129 months.

Despite the difficulty encountered during catheter removal in our patient, extraction was ultimately successful after meticulous dissection along the catheter tract combined with prolonged traction. This contrasts with the case reported by Mehra et al.,^3^ in which adhesion was so strong that removal resisted dissection along the catheter tract (up to its insertion point into the internal jugular vein) combined with traction as well as guidewire insertion. As both attempts at removing the catheter failed, the authors decided to cut the catheter and leave the distal end in situ to avoid the risk of vascular injury and potential hemorrhage, which would have worsened the patient’s prognosis. We support this approach, as retained catheter fragments adherent to the endovascular wall have not been associated with complications.^1^ This is further supported by Wilson et al.,^2^ whose single-center study showed no complications over a 6-year follow-up in patients with catheter fragments in situ, even without antithrombotic prophylaxis.

Nonetheless, endoluminal dilatation techniques such as Hong’s technique –designed to free “stuck” venous catheters using a balloon – have proven to be effective solutions in cases where the distal end of a catheter is strongly adherent to the endovascular wall. This method, which was refined by Quaretti et al.,^4^ is particularly useful in situations where the catheter must be cut and its distal end left in situ due to strong adhesion. One illustrative case involved a central venous dialysis catheter that had been implanted for 12 years and was successfully removed using this approach.^4^ This technique involves advancing a valved introducer sheath along the edge of the cut catheter near its entry point. Next, a rigid guidewire with a balloon catheter is inserted. Under fluoroscopic control, the balloon is inflated once it reaches the central venous lumen, releasing the distal end of the catheter from the endovascular wall of the central vein and allowing for its complete removal. In cases of abnormal catheter positioning, the rigid guidewire can be used to assist in repositioning the distal end of the catheter.

## CONCLUSION

Although TIVAD removal is a simple procedure, it can be complicated by rare cases of adhesion of the distal end of the catheter to the endovascular wall of a central vein. This phenomenon is favored by several known risk factors, such as prolonged dwell time, but it raises an important question: could an aberrant course of the catheter – particularly when the distal end is placed opposite to venous flow – also contribute to fibrin cuff formation and adhesion?

Finally, in cases where strong adhesion prevents TIVAD removal even after careful dissection and prolonged traction, the endoluminal dilatation technique appears to be the most effective option for releasing the distal end from the endovascular wall.
